# Is Virtual Reality Orientation Therapy Useful to Optimize Cognitive and Behavioral Functioning Following Severe Acquired Brain Injury? An Exploratory Study

**DOI:** 10.3390/brainsci14050410

**Published:** 2024-04-23

**Authors:** Rosaria De Luca, Andrea Calderone, Antonio Gangemi, Carmela Rifici, Mirjam Bonanno, Maria Grazia Maggio, Irene Cappadona, Isabella Veneziani, Augusto Ielo, Francesco Corallo, Angelo Quartarone, Davide Cardile, Rocco Salvatore Calabrò

**Affiliations:** 1 IRCCS Centro Neurolesi Bonino-Pulejo, S.S. 113 Via Palermo, C. da Casazza, 98124 Messina, Italy; 2 Department of Nervous System and Behavioural Sciences, Psychology Section, University of Pavia, Piazza Botta, 11, 27100 Pavia, Italy

**Keywords:** SABI, cognitive deficits, depressive symptoms, ROT (reality orientation therapy), virtual reality rehabilitation

## Abstract

Introduction: Severe acquired brain injury (SABI) is a leading cause of death and disability, and it is defined as a brain injury that occurs after birth due to traumatic or non-traumatic causes. Reality orientation therapy (ROT) uses repeated time–place–person orientation and meaningful stimuli to develop a better understanding of the environment and has great potential as an effective strategy to improve cognitive and behavioral functioning. Objective: This study aims to investigate the feasibility and potential effects of virtual reality orientation therapy (VR-rot) on optimizing cognitive and behavioral functioning and depressive symptoms post-SABI. Method: Forty patients with SABI were enrolled from October 2022 to December 2023 and divided into two groups: the experimental group (EG, *n* = 20) received VR_rot, while the control group (CG, *n* = 20) received standard ROT (S_rot). All patients were evaluated with a psychometric battery, including the Mini-Mental State Examination (MMSE) and the Hamilton Rating Scale for Depression (HRS-D), administered before (T0) and after the end (T1) of rehabilitation. Results: Within-group comparisons indicated a statistically significant change in MMSE scores from T0 to T1 in the EG and CG, with the EG showing a greater improvement than the CG. Regarding HRS-D scores, the EG showed a statistically significant change. VR-ROT could be a valuable tool for improving cognitive–behavioral functioning in SABI patients. Conclusions: The VRRS can help reduce depressive symptoms and improve the reality orientation deficit caused by traumatic brain injury and stroke on brain tissue. This study highlights the benefits of virtual reality.

## 1. Introduction

### 1.1. Severe Acquired Brain Injury

Severe acquired brain injury (SABI) is a leading cause of death and disability worldwide. SABI is defined as brain injury occurring after birth due to traumatic or non-traumatic causes, including ischemic or hemorrhagic stroke [1,2]. SABI patients must learn to live with reduced potential for physical, emotional, cognitive, and social functioning [3,4,5]. SABI also refers to neurological conditions due to acquired brain damage lasting more than 24 h with a score of 8 or less on the Glasgow Coma Scale (GCS) [6] and/or complex and severe neurological disorders that can only be treated in highly specialized intensive units [7]. In particular, SABI is a leading cause of disability and requires intensive rehabilitation treatment [8]. Depending on the location, extent, and depth of the brain lesion, patients with SABI show various cognitive–emotional changes. Memory, attention, and executive systems are predominantly affected [9,10,11]. Impulsivity and inhibition deficits are also frequently observed [12,13]. Over time, neurobehavioral and cognitive impairments have been shown to contribute more to overall disability after traumatic brain injury (TBI) than physical impairment [14]. The mental state of patients is associated with ‘higher brain dysfunction’, consisting of cognitive and cognitive–emotional integration [15]. Dysfunction is manifested by a range of symptoms including frustration, inappropriate emotional responses, and a lack of spontaneity [16]. Patients undergoing rehabilitation after TBI should be evaluated for mood disorders, which is a major cause of disability after TBI and a risk factor for poor recovery [17]. Treatments of TBI can be divided into pharmacologic and non-pharmacologic therapies. Non-pharmacological treatments include several rehabilitation therapies.

### 1.2. Cognitive Rehabilitation

Cognitive rehabilitation is a systematic, function-focused therapeutic activity based on understanding and assessing the patient’s brain and behavioral deficits. This type of intervention can be applied at all stages of post-injury recovery and in a variety of settings (e.g., hospital, outpatient, home environment). It can also be implemented in various ways (e.g., by individuals, families, groups) and by health professionals with different specialties (e.g., neuropsychologists, cognitive and occupational therapists, speech therapists) [18]. Typical cognitive training sessions are conducted individually or in groups [19,20,21] and facilitated by therapists and/or caregivers [22,23]. Sessions focus on specific cognitive skills training, and their duration depends on the individual’s tolerance and difficulty in performing the activity, taking into account a wide range of neurocognitive strengths and weaknesses. There are two main types of cognitive rehabilitation methods: traditional (paper-and-pencil exercises) and computer assisted (computer-based cognitive rehabilitation. Both methods use cognitive strategies to help patients improve their attention and concentration, visual processing, language, memory, reasoning, problem-solving, and executive functions [24,25].

### 1.3. Reality Orientation Therapy

The literature suggests that patients with SABI may experience temporal and spatial disorientation. Therefore, certain techniques should be employed to address this issue. For instance, reality orientation therapy (ROT) has great potential to improve cognitive functioning, disruptive behaviors, and emotional well-being in SABI patients. This technique involves repeated time–place–person orientation and meaningful stimuli to help the patient develop a better understanding of their environment and a sense of control. There are two forms of ROT: classroom ROT and 24 h ROT. Classroom ROT is led by a professional who engages the patient in orientation-related activities through structured courses and stimuli; 24 h ROT takes place in all situations where a professional or caregiver interacts with the patient during activities of daily living [26,27].

### 1.4. Virtual Reality

Virtual reality (VR) is a useful tool for cognitive rehabilitation. This type of training involves the patient interacting with a virtual or augmented environment through technology [28]. Virtual environments are computer generated and have special sensory characteristics that can be interacted with in real time [29]. The activities proposed to patients can offer forms of ecological and real-world demands of daily life, such as finding and purchasing objects to enhance brain plasticity and regenerative processes [30]. VR allows activities to be adapted to the patient’s abilities and performance to be monitored [31].

It also enables increased patient participation and motivation through auditory and visual feedback. For these reasons, this new technology may have advantages in treating patients; in fact, several studies have shown its effectiveness in cognitive improvement [30,31,32]. In the field of cognitive rehabilitation, the Virtual Reality Rehabilitation System (VRRS) is a commonly used tool. It is designed to facilitate neurological rehabilitation by using scenarios that simulate real-life situations through mediated simulation technologies. The VRRS has cognitive modules with different levels of difficulty that can improve the rehabilitation process and increase the recovery of cognitive abilities, reducing the frustration of neurological patients through different activities. [33]

Virtual reality orientation therapy (VR-ROT) is designed to stimulate the same cognitive areas as standard ROT but through the use of a virtual reality tool. No studies have been found in the literature investigating VR-ROT in patients with severe acquired brain injury by obtaining a single cognitive functioning score, but it is examined in a fragmented manner, not allowing a comprehensive and integrated view regarding the time, place, and orientation of the person. Furthermore, neuropsychological research in the treatment of depression demonstrates the therapeutic value of digital interventions based on playful activities by assessing the release of hormones such as endorphins and striatal dopamine responsible for feelings of pleasure and well-being during the patient’s interactive experiences with virtual reality [34,35].

In the light of this evidence, ROT would be implemented by the use of virtual reality, a comprehensive cognitive assessment, and by taking into account depressive symptoms. We believe that the use of VR-ROT could be feasible and at least not inferior to standard ROT in these patients’ populations.

The purpose of this study is to investigate the potential effects of VR-ROT on optimizing cognitive and behavioral functioning and depressive symptoms post-SABI.

## 2. Materials and Methods

### 2.1. Study Population

Forty subjects (mean ± SD age: 46.17 ± 15.02 years; 52.5% male) affected by SABI and attending the Neurorehabiliattion Unit of the IRCCS Centro Neu-rolesi “Bonino-Pulejo” between January 2022 and June 2023 were enrolled in the study and assigned to the experimental (EG: *n* = 20) or control (CG: *n* = 20) groups using simple random sampling. The sample size was selected based on resource availability, feasibility considerations within our clinical setting, and preliminary statistical power calculations to detect medium to large effects, appropriate for an exploratory study. A detailed description of the two groups is in Table 1. SABI patients and/or their caregivers were fully informed about the study and gave their cooperation and written informed consent. The study was conducted following the Helsinki Declaration of Human Rights, and the local Ethics Committee approved the study (IRCCS-ME-CE 08/21).

### 2.2. Procedures

Patients were enrolled according to the following criteria: (i) diagnosis of severe SABI (vascular or traumatic eziology) in the chronic phase (≥6 months from onset); (ii) presence of moderate to severe cognitive changes after SABI (i.e., MMSE ≥ 12); (iii) age range 18–70 years (iv) according to neuroradiological and clinical assessments; (v) good therapeutic compliance; (vi) absence of psychiatric illness (delirium, psychosis)

Patients were excluded if affected by (i) active epilepsy, (ii) disabling sensory impairment (including visual or hearing loss), (iii) severe medical illness (such as heart and pulmonary failure), (iv) severe cognitive or behavioral impairment that could potentially interfering with the training, (v) absence of the ability to understand verbal delivery of a simple order with a Token test score <4 and (vi) presence of debilitating behavioral alterations and severe psychiatric symptoms. Psychometric assessments included specific neuropsychological tests to assess cognitive functioning and behavioral performances. All study participants received ROT, in addition to the same psychological support and standard physical therapy. The VR-ROT group (20 SABI patients) received training to improve personal, temporal, and spatial orientation using the VRRS (24 sessions of 60 min each, three times a week for eight weeks), while the S-ROT group (20 SABI patients) received the same amount of standard ROT (24 sessions of 60 min each, three times a week for eight weeks) without the use of a virtual tool (for a detailed description, see Table 2 and Table 3).

### 2.3. Psychometric Measures

The psychometric measures included the MMSE, a neuropsychological test often used as a screening tool in the investigation of dementia and neuropsychological syndromes of different natures. The test consists of simple questions related to seven cognitive domains (temporal orientation, spatial orientation, verbal recording, attention and calculation, recall, language, and constructive application). Total scores range from a minimum of 0 to a maximum of 30 points, with scores below 18 indicating significant cognitive impairment, scores between 18 and 24 indicating moderate to mild impairment, 25 indicating borderline, and scores between 26 and 30 generally indicating normal cognitive functioning. However, it is necessary to clarify that an MMSE score between 26 and 30, in addition to other psychometric tests and clinical scales (the Clinical Dementia Rating Scale (CDR: 0.5), Montreal Cognitive Assessment (MoCA < 26); General Practitioner assessment of Cognition (GPCog ≥ 8), could indicate a pre-clinical condition, defined as mild cognitive impairment (MCI) [36]. However, due to calibration factors related to the subject’s age and educational background, this indicator is only a guide [37]. HRS-D is a clinical scale that assesses the presence or absence of depression symptoms. It is articulated in 21 items, including 4 items intended to subtype the depression, but which are sometimes incorrectly used to rate severity. HRS-D is characterized by a specific scoring, from not depressed with a score of 0–7 to a very severe depressive status (severe) with a score of >23 [38].

### 2.4. Standard ROT

The standard treatment focused on the orientation process, which was carried out by using paper and pencil along with other traditional tools and materials in a face-to-face approach between the therapist and patient. In fact, this approach is characterized by total human interaction between the SABI patient and therapist, realized in a traditional setting. Autobiographical, spatial, and temporal orientation are the main cognitive domains stimulated during a standard rehabilitative session realized in a dedicate room, administered in addition to psychological support and standard neurorehabilitation, including speech therapy and physiotherapy (see Table 2 and Table 3).

### 2.5. Virtual ROT

**Figure 1:**
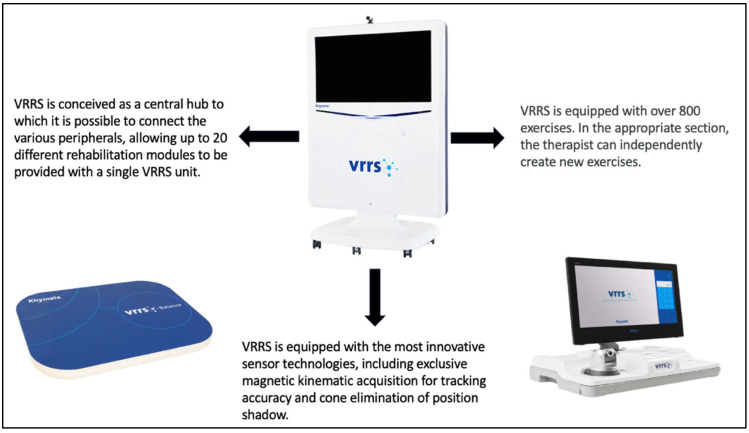
Description of the VRRS.

**Figure 2:**
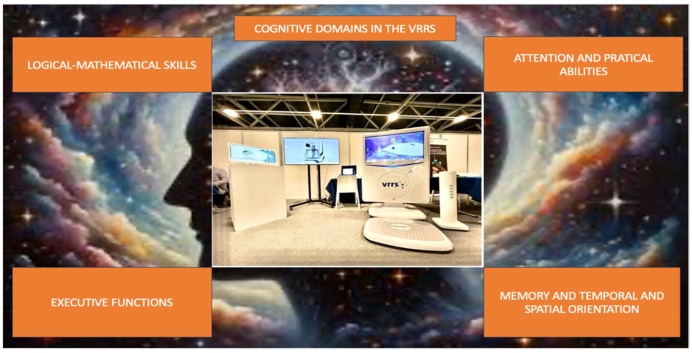
Cognitive domains present in the VRRS.

Virtual ROT training is also focused on strengthening the orientation process. It was developed to stimulate the same cognitive areas as the traditional standard ROT training group, but using a virtual interface, an innovative tool called the VRRS (Virtual Reality Rehabilitation System). It is divided into specific rehabilitative modules such as motor (posture, upper and lower limb activity, facial expressions, respiratory movements), logopedic, and cognitive (Figure 1). It is a valuable device that provides virtual exercises that are divided into specific cognitive domains (e.g., logical–mathematical skills, executive function, attention, etc.) (see Figure 2). The cognitive module included a dedicate sub domain focused on specific virtual tasks to stimulate the orientation of subjects with SABIs: personal, topographic, and temporal orientation. The virtual ROT program administered is reported in Table 2 and Table 3.

### 2.6. Statistical Analysis

Data were analyzed using Python 3.11.5, with statistical analyses performed using the SciPy library (version 1.11.1). A threshold of *p* < 0.05 was set for statistical significance. Descriptive statistics, including mean, standard deviation, and range, were calculated for each group to provide an overview of the dataset’s characteristics. Proportions were assessed by the Chi-squared test. The Shapiro–Wilk test was applied to assess the normality of distributions in MMSE and HRS-D scores at T0 and T1, guiding the selection of appropriate statistical tests. The MMSE scores at both T0 and T1 were not normally distributed, while the HRS-D scores were normally distributed. Consequently, non-parametric tests, specifically the Mann–Whitney U test for inter-group comparisons and the Wilcoxon signed-rank test for intra-group comparisons, were used for the MMSE scores. For the HRS-D scores, parametric tests, namely, the paired *t*-test for intra-group comparisons and the independent *t*-test for inter-group comparisons, were employed.

## 3. Results

**Figure 3:**
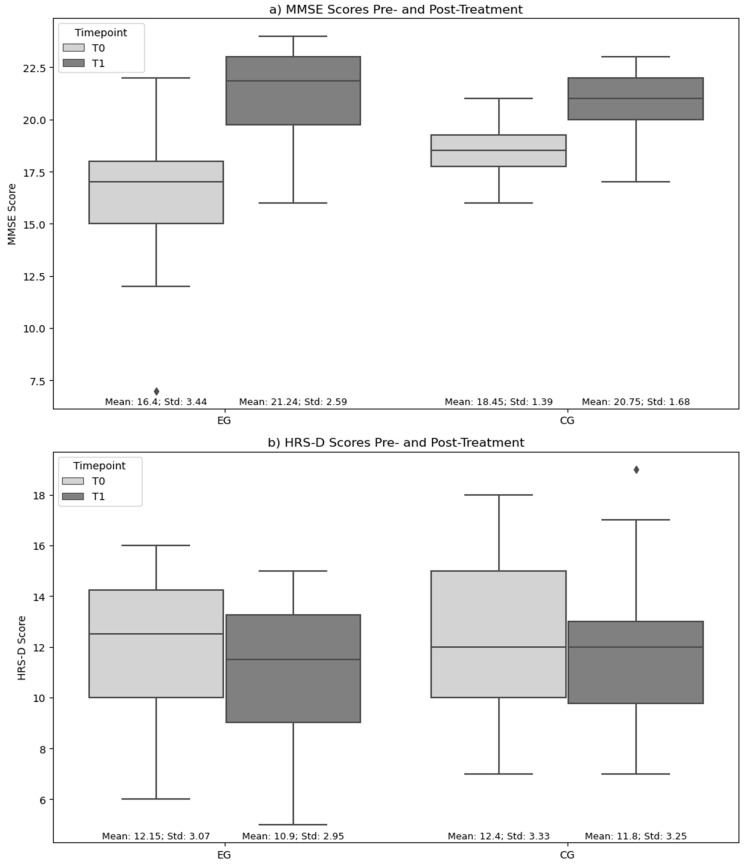
MMSE and HRS-D scores pre- and post-treatment. Legend: (**a**) MMSE scores; (**b**) HRS-D scores; EG: experimental group; CG: control group. Scores expressed as mean.

The EG and CG were comparable in terms of age, sex, and education level. Descriptive statistics indicated similar baseline characteristics between the two groups, with no significant differences in age (*p* = 0.84), education level (*p* = 0.32), and sex distribution (*p* = 1.00). Intra-group comparisons indicated a statistically significant change in MMSE scores from T0 to T1 within both the EG and the CG (*p* < 0.001), with the EG exhibiting a greater improvement compared to the CG (EG: from 16.4 ± 3.44 to 21.24 ± 2.59; CG: from 18.45 ± 1.39 to 20.75 ± 1.68), as shown in Figure 3. Regarding the HRS-D scores, the EG showed a statistically significant change (*p* < 0.001). In the inter-group analysis, the Mann–Whitney test for MMSE scores showed a statistically significant difference at T0 between the EG and CG (*p* = 0.021). For the HRS-D scores, no statistically significant differences were observed between the groups at baseline or post-treatment. The detailed results of the statistical analysis are reported in Table 4.

## 4. Discussion

As far we know, this is the first study that explored the potential effects of ROT applied using VR, focusing on orientation, the complex ability to locate oneself in one’s environment with reference to time, place, and people (including personal, temporal, and topographical sub-domains), in patients with SABI. Studies investigating VR-ROT by obtaining a single cognitive functioning score to evaluate treatment effectiveness could not be found in the literature. This lack of research may be due to the absence of advanced rehabilitation equipment in hospitals, which is often caused by high costs, accessibility problems, and a shortage of highly qualified therapists [39].

We found that SABI subjects receiving conventional ROT improved their global cognitive functioning and mood, although those treated with VR-ROT achieved better outcomes in MMSE and HRS-D’ scores. The results revealed that both groups showed a significant improvement in cognitive functioning, but it was greater in the experimental group. In addition, the experimental group alone also reported a significant improvement in depression.

The use of VR in the neurorehabilitation field has been increasing in the ten last years, demonstrating its efficacy in promoting cognitive [40] as well as motor recovery [41]. Kober et al., 2013 [42] suggested that the VR technique creates natural, customized, and complex simulated environments in which spatial deficits, such as disorientation and the ability to locate landmarks, can be examined in an accurate and ecologically sound manner. The current research is not uniform and investigates VR-ROT in patients with SABI in a fragmented manner, not allowing a comprehensive and integrated view regarding time, place, and person orientation. Recent studies have shown that virtual reality can improve spatial orientation in patients with stroke [43], traumatic brain injury, and other acquired brain injuries [44]. In fact, patients with Alzheimer’s disease who underwent virtual reality treatment showed significant improvements in spatial orientation when compared to traditional treatment [45]. These findings are consistent with our own study. Additionally, some authors have reported that virtual reality can improve cognitive performance in patients with cognitive impairment by using exercises related to personal history, identity, day, hour, and month [46], which is also in line with our own findings.

However, there are currently no studies available in the literature regarding personal and temporal orientation in patients with SABI through virtual reality. ROT provides for the continuous presentation of orientation information related to time, place, and person so that the individual can gain a greater understanding of their surroundings [47], allowing for improvements in cognitive function, disruptive behavior, and emotional well-being [48]. Limited studies in the literature that have focused on the effectiveness of ROT have concentrated more on neurodegenerative diseases. A recent meta-analysis [48] including 11 randomized trials showed that this therapy reported positive effects on cognitive function in patients with dementia. Other studies have also found cognitive benefits of ROT in elderly individuals [49], patients with dementia [50], and individuals with mild Alzheimer’s [45] and Parkinson’s disease dementia [51]. Additionally, the study by Onieva et al., 2018 [45] reported improvements in depressive symptoms in patients with mild Alzheimer’s disease. In line with our scores, other studies have confirmed an improvement in depression in patients with head trauma [31] and stroke [35] through virtual reality compared to conventional therapy. Neuropsychological research in depression therapy confirms the therapeutic value of digital interventions, highlighting the release of hormones such as endorphins and striatal dopamine, responsible for feelings of pleasure and well-being, during the patient’s interactive experiences with virtual reality [34].

Overall, the results of our study are consistent with existing research on VR and its impact on cognitive functioning in patients with SABI. Virtual reality has numerous advantages over conventional therapies [52]. The first advantage of using virtual reality in the rehabilitation of patients with SABI is the creation of various artificial environments similar to real ones that the individual has to interact with through sight, hearing, and touch [53]. Through virtual scenarios, the central nervous system receives more sensory feedback (auditory, visual, tactile), which can create changes in synaptic plasticity and enhance learning [54]. Additionally, virtual reality provides a safe and harmonious environment with unlimited repetitions of the same task selected according to the patient’s specific characteristics [52]. The subject immersed in the illusory world can better handle mistakes and reproduce exercises as compared to the repetitive tasks involved in traditional therapeutic rehabilitation [55]. This facilitates improvement through trial-and-error procedures and allows for a reduction in stress and greater frustration tolerance [39]. The therapist can propose exercises through virtual reality that are expensive, difficult, or impossible to perform in conventional practice [55] such as planet exercise, which generates enthusiasm and increases motivation and adherence to treatment [54,55]. Furthermore, the degree of interaction with the digital tool can be so high that it modifies the patient’s reaction to pain, with an attenuation of discomfort during the virtual experience [56,57].

In addition, the use of brain–computer interfaces (BCIs), which record and decode activity in real time by providing sensory feedback through an avatar in a virtual reality environment, has brought benefits to neuroplasticity by facilitating the learning and rehabilitation of impaired cognitive functions [58,59]. Although various hardware and software can be expensive, there are low-cost portable VR options that can be installed in commonly used smartphones [60,61]. These options offer inexpensive and practical ways to use virtual reality through smartphones that many consumers already own. Although they do not offer individuals the same quality and functionality as more expensive solutions, they are a good starting point for offering cognitive rehabilitation methods that can be applied in the home environment [61].

Compared to studies that support virtual reality-based rehabilitation, a recent study [62] assessed the effect of virtual reality-based therapies on global and domain-specific cognition after stroke, concluding that VR rehabilitation is not superior when used to control interventions in improving global and domain-specific cognition in stroke patients. However, their conclusions were only based on data from two studies, resulting in insufficient statistical power.

Virtual reality can cause adverse reactions such as cyber-sickness, a type of kinetosis resulting from virtual immersion. Subjects present vomiting, pallor, drowsiness, nausea, belching, salivation, disorientation, headaches, dizziness, postural instability, and illusion of movement. In addition, prolonged use can cause headaches and visual fatigue that tend to be mild and temporary [63].

This study emphasizes an integrated approach through VRRS-ROT in patients with SABI and focuses on orientation, the ability to locate oneself in one’s environment with reference to time, place, and people. Given the positive results in cognitive functioning and in depression, VRRS-ROT could be incorporated into clinical practice because (i) the engaging and rewarding experience reduces frustration and increases motivation and adherence to therapy; (ii) it allows, even through a single exercise, several cognitive areas to be worked and joint improvements to be achieved; (iii) it allows data from each session to be saved, which is useful for ongoing monitoring essential in clinical practice. This practice could be used for the design of future interventions and therapeutic approaches to improve patients’ spatial, temporal, personal, and emotional orientation skills, thus contributing to their overall well-being and improved quality of life.

Our study has its limitations, and we have taken note that the sample size is too small to extend the results to the general SABI population; however, the study was conceived as an explorative study, and the number of subjects enrolled are in line with the study design. Another limitation was the lack of follow-up and the use of too-generic scales to investigate the ROT domains. More studies with larger samples, higher-quality methodology, and long-term follow-up are needed to confirm the therapeutic effect of VR on cognitive and behavior outcomes. Nonetheless, this is an exploratory study aimed at investigating the feasibility and potential effect of ROT applied using VR in patients with SABI. Then, more specific tests are needed to confirm the positive changes observed though the administration of the MMSE. The objective of this exploratory research is to gather preliminary information that will help define the frequent problem of disorientation post-SABI, suggesting the hypotheses of a promising use of VR-ROT, in additional to conventional neurorehabilitation.

## 5. Conclusions

We have showed the promising feasibility and potential positive effect of ROT applied using VR in patients with SABI. Our research has shown that VRRS is a valuable tool for optimizing cognitive–behavioral functioning in patients with SABI. VRRS can help reduce depressive symptoms and improve the reality orientation deficit caused by traumatic brain injury and stroke on brain tissue. Due to the patient’s deep involvement in a virtual environment, VRRS increases motivation and treatment adherence through sounds, colors, and environments experienced as real. Furthermore, it can help improve their confidence in daily challenges through safe virtual situations that simulate activities they might encounter in the real world. In conclusion, virtual reality is an important therapeutic resource that can be used to improve the well-being of patients with spatial, temporal, and personal orientation deficits through practical and personalized experiences.

The primary limitation of the study is attributed to the small sample size. Having only 20 patients in each group undermines the ability to generalize the outcomes of the study to the entire population of patients with SABI.

Our results serve as a preliminary study that should lead to larger randomized controlled clinical trials and long-term follow-up to thoroughly evaluate the benefits of virtual reality.

Although the potential of VRRS in patients with SABI is not yet fully explored, this study has shown that VR is a valuable rehabilitation tool that produces positive results and improves quality of life.

## Figures and Tables

**Table 1:** Socio-demographic description of the sample.

	All	EG	CG	*p*-Value
Participants	40	20 (50.0)	20 (50.0)	-
Male	21 (52.5)	10 (50.0)	11 (55.0)	1.00
Age (years)	46.17 ± 15.02	47.30 ± 14.03	45.05 ± 16.23	0.64
Education (years)	10.15 ± 3.11	10.65 ± 2.98	9.65 ± 3.23	0.32

Legend: Experimental group (EG); control group (CG). Continuous variables are expressed as mean ± standard deviation, whereas categorical variables are expressed as frequencies (percentages).

**Table 2:** ROT training for SABI: individual session, duration, and type of treatment.

Rehabilitation Program	Intervention	Individual Session Duration	Type of Intervention	Exercise Time	Cognitive Domains
3-month standard approach, paper and pencil, face to face	S-ROT + Psychological support	3 weekly sessions of 60 min	S-ROT (45 min)	15 min 15 min 15 min	Personal/autobiographical orientation Temporal orientation Spatial orientation
			Psychological Support	15 min	Motivation and mood
3-month virtual approach VRRS interface	VR-ROT + Psychological support	3 weekly sessions of 60 min	V-ROT (45 min)	The same exercise time as the conventional ROT training group.	The same cognitive domains as the conventional ROT training group.
			Psychological Support		

**Table 3:** ROT program for people with SABIs: personal, spatial, and temporal orientation.

Domain	Sub Domains	Short Description	Standard Tasks Paper-and-Pencil Approach— Human Interface	Virtual Activities Virtual-PC-Based Interface
ORIENTATION	Personal Orientation	The capacity to combine information related to our personal history and identity, including our age, civil status, or education level.	Activities that require patients to answer a series of questions about their personal life have been designed to rehabilitate personal orientation, with varying levels of difficulty and assistance depending on the patient’s specific needs. The objective is to observe and select the usual stimuli that are given, which may include photos (of friends, pets, etc.) that are emotionally meaningful for patients. Audio–video materials, like voice recordings of family, friends, and colleagues, can also be used to listen to emotional–meaningful songs or to observe preferred scenes from favored movies or videos about personal life events (such as childbirth, significant events, etc.).	Virtual activities have been developed to improve personal orientation by requiring patients to answer a series of questions about their personal life. The patient’s specific needs can affect the level of difficulty and assistance provided for these virtual tasks. Emotional virtual images, such as personal settings or biographical virtual photos (about their home, wife, mother...), can be viewed and selected. Using VRRS integrated to the virtual system, patients can listen to to affective audio–video materials such as voice recordings of family members, music tracks that are emotionally meaningful, a main list of favorite movie scenes, and videos of personal life scenes (birth of children, significant personal events in life).
	Spatial Orientation	The capacity to manage information concerning one’s starting point, current location, destination, etc.	In the program for spatial orientation, patients were taught to connect different objects to the places where they can be bought and the professionals who work there through exercises. The aim was to promote spatial awareness by recalling memories of places, cities, or streets, using impromptu paper-and-pencil materials. This involved conducting visual–spatial tasks and spatial awareness exercises, resolving traditional puzzles, identifying the location of 2D objects (center, right–left), drawing activities, and recognizing shapes and spatial relationships.	Spatial orientation was addressed in the virtual program by incorporating virtual exercises that involved patients connecting objects to places where they can be purchased and the professionals who work there. In order to foster spatial orientation in a virtual environment and promote topographic sense and perception, virtual reasoning activities can be used to recognize places, cities, and different locations. This can include managing virtual orientation tasks, completing spatial awareness activities by completing virtual puzzles or adjusting the position of virtual elements (center, right–left), and exploring interactive maps and shapes through virtual drawing or painting.
	Temporal Orientation	The ability to keep track of information about various events or situations and arrange it in chronological order. It encompasses information related to the day, time, month, year, and the moment of engaging in certain behaviors, holidays, seasons, etc.	Tasks that require patients to provide information on the time, month, and seasons of the year are designed to improve temporal orientation. Managing information about days, times, and months can be achieved by using the repetition and recall of specific information, like personal data and events, in conjunction with face-to-face activities to increase temporal orientation abilities. SABI patients are required to provide the time, day, month, year, and current season in this activity, with the option to select the month they are currently in during the exercise.	Virtual tasks are available to patients to improve their temporal orientation by requiring them to tell the time, month, and season of the year. Increasing temporal orientation abilities can be achieved by repeating and recalling specific information, such as personal data and personal events; managing information related to days, times, and months; and utilizing VVRs and virtual environments. VR is used to continuously transmit information through visual, written, or auditory modes.

**Table 4:** Intra- and inter-group analysis between EG and CG.

		EG	CG	
		Median (1st Qu.–3rd Qu.)	Median (1st Qu.–3rd Qu.)	*p*-Value
MMSE	T0	17.00 (15.00–18.00)	18.5 (17.75–19.25)	**0.021**
	T1	21.85 (19.75–23.00)	21.00 (20.00–22.00)	0.251
	*p*-value	**<0.001**	**<0.001**	
HRS-D	T0	12.50 (10.00–14.25)	12.00 (10.00–15.00)	0.806
	T1	11.50 (10.00–16.00)	12.00 (9.75–13.00)	0.366
	*p*-value	**<0.001**	0.124	

Legend: Experimental group (EG); control group (CG); Mini Mental State Examination (MMSE); Hamilton Rating Scale—for depression (HRS-D). Significant differences are in bold.

## Data Availability

The data that support the findings of this study are not openly available due to reasons of sensitivity and are available from the corresponding author upon reasonable request.
